# Solid Pseudopapillary Neoplasm of the Pancreas: A Comprehensive Review Focusing on the Role of Endoscopic Ultrasound-Guided Radiofrequency Ablation as an Alternative Treatment

**DOI:** 10.3390/cancers17132240

**Published:** 2025-07-04

**Authors:** Tawfik Khoury, Moaad Farraj, Wisam Sbeit, Andrea Lisotti, Bertrand Napoléon

**Affiliations:** 1 Department of Gastroenterology, Galilee Medical Center, Nahariya, Faculty of Medicine in the Galilee, Bar-Ilan University, Safed 5290002, Israel; wisams@gmc.gov.il; 2 Department of Surgery B, Galilee Medical Center, Nahariya, Faculty of Medicine in the Galilee, Bar-Ilan University, Safed 5290002, Israel; farrajm@gmc.gov.il; 3 Gastroenterology Unit, Hospital of Imola, University of Bologna, 33-40126 Bologna, Italy; a.lisotti@ausl.imola.bo.it; 4 Department of Gastroenterology, Hôpital Privé Jean Mermoz, Ramsay Santé, 69008 Lyon, France; dr.napoleon@wanadoo.fr

**Keywords:** solid pseudopapillary neoplasm, pancreas, treatment, EUS, RFA

## Abstract

**Background:** Solid pseudopapillary neoplasm (SPN) is a rare pancreatic tumor with malignant potential. Its diagnosis has grown alongside increased use of abdominal imaging. SPN is suspected after classical findings in abdominal imaging studies; however, endoscopic ultrasound-guided (EUS) fine needle aspiration can support preoperative diagnosis. The treatment of choice is still surgical intervention, with an intent to reach curative resection. The prognosis is excellent. Recently, emerging data on EUS-guided radiofrequency ablation (RFA) suggest changing the choice of treatment for small SPN. **Methods:** We provide a comprehensive overview on pancreatic SPN with a focus on treatment, adverse events, recurrence rate, and outcomes. In addition, we provide a literature summary and pool data analysis. **Results:** Overall, 70 papers including 6651 patients were identified. The mean SPN size was 5.8 cm, metastasis rate was 1.9%, and recurrence rate was 3%. Moreover, the mortality rate was low at 0.2%, although high postoperative adverse events were reported (32.4%). Small SPN (<2 cm) was present in 4.1% of the studies. Two studies reported EUS-RFA for small SPN <2 cm, without recurrence at a median follow-up of 18.5 months. **Conclusions:** SPN still necessitates surgical intervention given its malignant potential. However, EUS-RFA can represent a promising and safe therapeutic option for SPN < 2 cm.

## Simple Summary

Solid pseudopapillary tumor (SPN) of the pancreas is an uncommon tumor associated with malignant transformation potential and frequently requiring surgical intervention for definite cure. This study reviewed and analyzed the available evidence on the treatment outcomes and recurrence rate of SPN. Results from 70 studies involving 6651 patients showed a nonnegligible metastatic rate at presentation and considerable perioperative morbidity. There is an emerging role of endoscopic ultrasound guided radiofrequency ablation for small SPN.

## 1. Introduction

Solid pseudopapillary neoplasms (SPN) were first described by Frantz in 1959 and are considered cystic pancreatic neoplasms. SPNs are known by many names, including Frantz tumor, papillary cystic tumor, and solid and papillary epithelial neoplasm. SPNs are frequently diagnosed because they are found in more than 2% of routine imaging exams performed due to another causes [1]. Most SPNs occur in young women (approximately 80% of cases) in their second or third decade of life [2,3]. They are located in the body and tail of the pancreas in approximately two-thirds of patients; however, they can arise in the pancreatic head [3]. They are considered low-grade malignant neoplasms composed of both solid and cystic components with pseudopapillary areas [4]. The estimated prevalence of this tumor is growing due to the improvement of imaging techniques. It is estimated to account for 1% to 3% of all pancreatic tumors [5]. Radiology is the cornerstone of SPN diagnoses. For bigger lesions, ultrasound with contrast and computed tomography (CT) with injection shows a heterogeneous lesion due to its solid and cystic components (secondary to hemorrhagic involution of the tumor) and a hyperenhanced rim [6,7]. Definitive diagnosis is obtained via histological analysis of the lesion. The diagnosis is usually made after the resection of the tumor. In this case, surgery is proposed to patients with a high suspicion of SPN, given clinical and radiological characteristics. But the accuracy of this diagnosis strategy is imperfect. Kim et al. [8] reported that only 46% of patients were correctly diagnosed with SPN according to radiological and clinical characteristics. Endoscopic ultrasound-guided fine needle aspiration (EUS-FNA) is a reliable tool to achieve a definitive diagnosis of SPN. It is safe and can provide histocytological specimens preoperatively, which can guide a targeted surgical approach; its accuracy is estimated to be between 75% and 83% [9]. Although the risk of adverse events is quite low, the risk of tumor spreading, especially peritoneal tumor spreading, cannot be ruled out. A recent retrospective meta-analysis by Karsenti et al. [10] of 149 SPN did not show a significant risk of tumor spreading after EUS-FNA; the only risk factor with recurrence was older age. Due to its malignant potential, the prognosis of this tumor can be quite severe. Therefore, the gold standard therapy is surgical resection. Nonetheless, such an aggressive surgery can be quite invasive and have severe side effects; surgeons now tend to propose conservative surgery, especially for younger patients [11,12]. In these cases, endoscopic ultrasound-guided radiofrequency ablation (EUS-RFA) should be an alternative in pancreatic neuroendocrine tumor treatment [13]. In this updated review, we summarize the available literature on SPN, focusing on treatment, adverse events, and recurrence rate following treatment.

### 1.1. Tumor Characteristics

SPNs are quite rare and account for 3% of cystic pancreatic neoplasms [14]. Macroscopically, SPNs are generally well known for their large diameter, with a mean size of 8.6 ± 4.3 cm according to a previous study [3]. In extremely rare cases, these tumors can occur outside of the pancreas, i.e., in the retroperitoneum, liver, digestive tract, omentum, ovary, or lungs [15]. Morphologically, an SPN appears as a large solid tumor with cystic components in 60% of cases, representing an area of hemorrhage and necrosis, calcifications (15–20% of cases), and a rim of fibrous and hypervascularized capsule [16]. SPNs can also be only cystic (in 25% of cases) or tissular (15% of cases) [3,7,8].

Histologically, SPNs are characterized by the presence of hyaline globule, which is found in about 5% of pancreatic neuroendocrine tumors [17]. Immunostaining of SPNs is positive to numerous proteins, but beta-catenin and E-cadherin are more specific and sensitive. Sensitivity and specificity of beta-catenin was 98.9% and 97%, respectively [18], whereas a Ki67 index > 4% is significantly associated with worst prognosis and recurrence [19]. On the other hand, E-cadherin, a cell adhesion molecule, is typically lost or reduced in SPN. This molecular pattern of E-cadherin loss, along with abnormal beta-catenin expression, is a typical feature of cystic pancreatic neoplasms and is thought to contribute to the tumor’s growth pattern and tendency for cystic degeneration [20].

### 1.2. Clinical Behavior and Diagnosis

SPNs can be asymptomatic or produce symptoms such as palpable abdominal mass, abdominal pain or discomfort, bloating, nausea, vomiting, and weight loss. Some symptoms can be quite severe, such as jaundice and pancreatitis [21].

As previously noted, radiology is a cornerstone of SPN diagnosis. With bigger lesions, ultrasound with contrast and CT with injection can show a heterogeneous lesion due to its solid and cystic components (secondary to hemorrhagic involution of the tumor) and hyperenhanced rim. Small SPNs are purely solid lesions. During the pancreatic phase, there is weak enhancement compared to the surrounding pancreatic parenchyma; in the hepatic venous phase, there is gradual increase in enhancement. CT is superior to ultrasound because it can find extrapancreatic lesions (primitive or metastatic) and study vascular and lymph node invasion [6,7]. Definitive diagnosis is obtained via histological analysis of the lesion. The diagnosis is usually made after tumor resection. In this case, surgery is typically proposed to patients with a high suspicion of SPN based on clinical and radiological characteristics. But the accuracy of this diagnosis strategy is imperfect. Kim et al. [8] reported in their study that only 46% of patients were correctly diagnosed with SPN according to radiological and clinical characteristics.

EUS-FNA is a reliable tool to achieve a definitive diagnosis of SPN. It is safe and can provide histocytological specimens preoperatively, which can guide a targeted surgical approach; its accuracy is estimated to be between 75% and 83% [9]. Although the risk of adverse events is quite low, the risk of tumor spreading, especially peritoneal tumor spreading, cannot be ruled out but seems exceptional. A recent retrospective meta-analysis by Karsenti et al. [10] of 149 SPNs did not show a significant risk of tumor spreading after EUS-FNA; the only risk factor with recurrence was older age.

### 1.3. Surgical Treatment of SPN

Due to the malignant potential of pancreatic SPN, en bloc surgical resection is the treatment of choice. As patients are typically young when diagnosed and prognosis is good after treatment, organ preservation is advocated if feasible [22]. Thus, tissue-sparing surgery with fewer long-term complications, such as endocrine and exocrine pancreatic insufficiency, is preferable [22]. The type of surgery depends on the lesion location. For lesions of the corpus and tail of the pancreas [22], distal pancreatectomy with spleen preservation is preferred when there is no splenomegaly or vascular (splenic artery and vein) and hilar invasion. Central pancreatectomy with pancreatico-jejunostomy or pancreatico-gastrostomy is preferred for SPN of the pancreatic neck. Pylorus-preserving pancreaticoduodenectomy is the surgery of choice for lesions in the head of the pancreas. Pylorus preservation is associated with a decrease of dumping syndrome, delayed gastric emptying, and diarrhea. Although surgical resection is the preferred approach, for SPNs with diameters up to 20 mm, enucleation can be a treatment option to avoid pancreatic resection and better preserve exocrine and endocrine pancreatic function [23].

## 2. Methods

We performed a comprehensive search of MEDLINE, PubMed, and Embase databases from 1 January 1996 to 1 January 2025. The search was limited to English-language publications and independently conducted by two reviewers (Tawfik Khoury and Moaad Farraj). Inclusion criteria were: (a) population: studies reporting on solid pseudopapillary tumor of the pancreas, pancreatic solid pseudopapillary tumor, or Frantz tumor; (b) intervention: surgical intervention or EUS-RFA; and (c) outcome: surgical outcome and recurrence rate. Exclusion criteria were papers reporting on fewer than 10 patients, studies omitting data on surgical outcomes and recurrence rates, and abstracts not published in a peer-reviewed journal.

A total of 1588 abstracts were identified and examined. Overall, we identified 70 relevant papers, which were fully accessed, analyzed, and discussed.

### 2.1. Evidence in the Literature

Table 1 demonstrates the characteristics of all included studies. The largest cohort was reported by Chen et al. [24], which included 486 patients. In their study, SPNs had a mean size of 5 cm. Six patients (1.2%) had metastatic disease on presentation. Most patients underwent surgical resection (89.7%). Notably, 20 patients (4.1%) had recurrence at a median follow-up of 58.5 months. None of the patients had SPNs less than 2 cm. Another large study was reported by Liu et al. [25] and included 454 patients. The mean SPN size was 5.4 cm, and 14 patients (3.1%) had evidence of metastasis on presentation. In this study, 451 patients (99.3%) underwent surgery (pancreaticoduodenectomy for 50 patients, pylorus-preserving pancreaticoduodenectomy for 31 patients, distal pancreatectomy for 104 patients, distal pancreatectomy with splenectomy for 131 patients, and local resection for 135 patients). Follow-up data were available for 390 patients, and 16 (4.1%) had recurrence at a median follow-up of 66 months. Of note, the morbidity rate was 16.9% (76 patients). Additionally, a large study by Lee et al. [26] reported retrospectively on 375 patients with mean size of SPN of 4.6 cm and low metastasis rate at presentation of 1.1%. Notably, the recurrence rate was 2.1% at a median follow-up of 39 months. After combining data across the 70 included studies (6651 patients), overall, 98.1% of patients underwent surgical treatment. The mean size of SPN was 5.8 cm, the rate of metastasis was 1.9%, and the mortality rate after surgery was 0.2%. The recurrence rate was 3% in a median follow-up period of 51.7 months. Only 5.8% of patients underwent EUS-FNA, and 4.1% had an SPN lesion size of ≤2 cm. Table 2 provides a data summary of our review. Moreover, regarding adverse events reported in the included studies, 50 studies reported postoperative morbidity, including 1163 adverse events involving 3587 patients (32.4%). Pancreatic fistula was the most common adverse event, occurring in 559 cases (48.1%), followed abscess or abdominal collections (6.9%) and wound or abdominal infection (6.3%). Notably, pancreatic insufficiency was reported in 4.6% of patients. Table 3 details the adverse events reported in the included studies. Our results were in line with a previous systematic review [3], including 2744 patients that showed a recurrence rate of 4.4%, while the mean tumor size was higher (8.6 cm), compared to 5.8 cm in our review. This might be due to the easily available and widespread use of abdominal imaging which led to earlier diagnosis of SPN. Similarly, a recent systematic review by Mazzarella et al. reported on 1384 patients; in total, 99.3% of them underwent surgery, with a recurrence rate of 2.8% at a mean follow-up of 4.2 years [27]. To the best of our knowledge, this is the largest review reporting 6651 patients. Therefore, our analysis might reinforce the evidence on the treatment and outcome of pancreatic SPN.

### 2.2. Metastatic SPN at Presentation

Overall, 29 studies reported metastatic SPN involving 124 patients. The most common site for SPN metastasis was the liver (41 patients, 33.1%), followed by spleen metastasis (six patients, 4.8%). Most studies did not report the patients’ age, only 10 studies reported the mean size of SPN (8.5 cm), and only seven studies reported the location of the SPN (pancreatic head was the most common site). No SPN ≤ 2 cm was reported with metastasis, but details were not noted in every trial. Interestingly, no lymph node metastasis was described. Table 4 demonstrate the study characteristics of the metastasized SPN.

### 2.3. Future Directions

In recent years, interest has been growing in EUS-RFA for pancreatic lesions due to the thermal sensitivity of the pancreatic gland. RFA applies an alternative current to the tumor that allows thermal necrosis of the surrounding tumoral tissue.

EUS-RFA has recently emerged as a therapy for neuroendocrine tumors [13], pancreatic cystic neoplasm (mainly intraductal papillary mucinous neoplasm), or locally advanced and nonresectable pancreatic carcinoma, with the main advantages of this technique being its effectiveness and low morbidity [94]. One limit of RFA in neuroendocrine tumor treatment is the absence of lymph node resection. This risk is not present in SPN because metastases have only been described as affecting other organs (mainly the liver). A recent study involved three patients with SPN treated with EUS-RFA. The mean size of SPN was 16.7 mm. Notably, all three had pretreatment EUS-FNA. None of them had procedure-related adverse events or developed recurrent disease at a median follow-up of 9 months [95]. In another recent study, Choi et al. [96] reported on two patients with SPNs of 23 and 20 mm who were successfully treated with EUS-RFA; the patients without complications or recurrence at a median follow-up of 28 months after treatment. Given this information, the morbidity and risk of pancreatic fistula in pancreatic surgery, even in conservative surgery, coupled with the known safety of EUS-RFA and its effectiveness in several pancreatic lesions, means EUS-RFA for SPN could be a promising alternative therapeutic option in patients with SPNs less than 2 cm. However, these data are preliminary in nature and reflect very small samples (see Table 5), so more prospective multi-institutional studies with longer follow-up are needed to confirm its safety and efficacy. Still the main treatment of pancreatic SPN is surgery. According to the authors’ expertise, coupled with the very limited data, we suggest EUS-RFA in inoperable patients with SPNs, preferrable up to 2 cm in size, and 2 mm distance from main pancreatic duct.

## 3. Conclusions

SPNs are rare pancreatic tumors, but with the advancement of medical imaging, their incidence is increasing, and the size of detected lesions is decreasing year by year. The standard of care is to propose surgery to every patient, including conservative surgery when feasible.

In this review of 70 papers involving 6651 patients, we determined that most patients underwent surgical resection because it is acceptable worldwide to perform surgery for SPN regardless of tumor size due to its malignant transformation potential. To date, the treatment of choice for SPN is still surgical management [98]. However, this approach is associated with postoperative morbidity and a high rate of adverse events (32.4%); the most common adverse event was postoperative pancreatic fistula (48.1%), primarily occurring after central pancreatectomy. Therefore, it is preferable to perform surgery at medical facilities with experienced practitioners [99]. Additionally, previous data indicated a high recurrence rate ranging from 6% to 14% [100,101]. In our comprehensive review involving a large number of patients, the recurrence rate was 3%, and the mean SPN size was 5.7 cm among patients who experienced recurrence. Therefore, further studies are needed to elucidate predictors of SPN recurrence. Finally, the promise of EUS-RFA for SPN seems to be gaining more attention, especially for small SPNs. Given the generally young age of patients at diagnosis, morbidity rate related to surgery, absence of risk of metastatic lymph nodes, and possibility of recurrence even after complete resection, a more conservative treatment might be recommended for localized SPNs < 2 cm. EUS-RFA has already shown its effectiveness in neuroendocrine tumors, and it has low morbidity. This technique is tissue sparing and less likely to induce endocrine or exocrine insufficiency than surgery, even conservative pancreatic surgery. Therefore, more data are needed regarding this novel treatment option.

## Figures and Tables

**Table 1:** Characteristics of the included studies.

Citation	Study Type	Patients, *N*	Size of SPN (cm)	Metastasis, *N* (%)	Treatment	Morbidity After Surgery	Mortality After Surgery	Location, *N*	Recurrence, *N* (%)	Follow-Up, Median, Months	EUS-FNA, *N* (%)	Lesion ≤ 2 cm, *N*
Sun et al. [28]	Retrospective	118	5.9	0	Surgery	NR	1 (0.8)	Head (33) Neck (19) Body, tail (66)	2 (1.7)	59.2	10 (8.5)	NR
Liu et al. [25]	Retrospective	454	5.4	14 (3.1)	Surgery	76 (16.9) ^NR^	0	Head, neck (161) Body, tail (115) Other (72)	16 (4.1)	66	18 (4)	NR
Zou et al. [29]	Retrospective	62	5.4	1 (1.6)	Surgery	26 (41.9) ^A^	0	Head (62)	0	46	0	NR
Gao et al. [30]	Retrospective	194	5.1	0	Surgery	129 (66.5) ^B^	0	NR	3 (1.5)	31	0	NR
Chen et al. [24]	Retrospective	486	5	6 (1.2)	Surgery (89.7%)	NR	NR	Head (186) Body, tail (285) Diffuse (1) Unknown (8) With metastasis (6)	20 (4.1)	58.5	0	0
Tasar et al. [31]	Retrospective	23	4	0	Surgery	9 (39) ^C^	1 (4.3)	Head (9) Body (6) Tail (8)	0	53	0	NR
Oase et al. [32]	Retrospective	28	5.4	0	Surgery	11 (39.3) ^D^	0	Head (12) Neck (1) Body (2) Tail (13)	2 (7.1)	41	17 (60.7)	NR
Shyr et al. [33]	Retrospective	31	5.7	0	Surgery	9 (29) ^E^	0	Head (14) Neck (1) Body (6) Tail (10)	1 (3.2)	67.7	0	0
Dhali et al. [34]	Retrospective	28	9.03	0	Surgery	22 (78.6) ^F^	1 (3.5)	Head (8) Body (8) Body, tail (10) Tail (2)	0	36	12 (42.8)	0
Li et al. [35]	Retrospective	166	4	0	Surgery	33 (19.9) ^G^	1 (0.6)	Head (69) Body (19) Tail (17) Multiple (64)	6 (3.6)	49	0	0
Wei et al. [36]	Retrospective	221	4.8	0	Surgery	84 (38) ^H^	0	Head (90) Body (24) Tail (23) Diffuse (84)	10 (4.5)	54	0	NR
Zhang et al. [37]	Retrospective	39	4.8	0	Surgery	17 (43.6) ^I^	0	Head (8) Neck (12) Body, tail (19)	1 (2.6)	24	3 (7.7)	NR
Wang et al. [38]	Retrospective	22	5.1	0	Surgery	NR	0	Head (1) Body (9) Tai (12)	0	69	7 (31.8)	2 (9.1)
Paredes et al. [39]	Retrospective	74	7.9	0	Surgery	45 (60.8) ^J^	1 (1.4)	Head (32) Body (16) Tail (26)	6 (8)	40.2	0	NR
Wang et al. [40]	Retrospective	63	4.9	0	Surgery	13 (20.6) ^K^	0	Head, neck (25) Body, tail (38)	2 (3.2)	58	0	NR
Rathi et al. [41]	Retrospective	13	7.7	0	Surgery	5 (38.5) ^L^	0	Head (2) Body, tail (7) Tail (4)	1 (7.7)	42	0	NR
Jena et al. [42]	Retrospective	15	6.4	0	Surgery	8 (53.3) ^M^	1 (6.7)	Head (2) Body (3) Tail (5) Body, tail (5)	0	59	6 (40)	NR
Kim et al. [43]	Retrospective	51	7	0	Surgery	39 (76.5) ^N^	0	Head (16) Body (9) Tail (26)	3 (5.9)	69	NR	NR
Kim et al. [44]	Retrospective	98	4.6	0	Surgery	29 (29.6) ^NR^	0	Proximal (29) Distal (69)	10 (10.2)	NR	0	NR
Chen et al. [45]	Retrospective	63	7	0	Surgery	NR	0	Head (16) Body (5) Tail (17) Body, tail (22 Outside (3)	9 (14.3)	42	0	0
Yang et al. [46]	Retrospective	193	5.2	3 (1.6)	Surgery	NR	NR	Head, neck (93) Body, tail (100)	7 (3.6)	53	0	NR
Lee et al. [26]	Retrospective	375	4.6	4 (1.1)	Surgery	NR	NR	Head (114) Body, tail (261)	8 (2.1)	39	0	63 (16.8)
Kotecha et al. [47]	Retrospective	22	4.5	0	Surgery	NR	0	NR	0	127.9	10 (45.5)	NR
Uğuz et al. [48]	Retrospective	24	5.8	0	Surgery	6 (25) ^NR^	0	Head (9) Body (4) Tail (11)	NR	60	21 (91.7)	NR
Song et al. [49]	Retrospective	102	6.6	0	Surgery	30 (29.4) ^O^	0	Head, neck (36) Body, tail (65) Outside (1)	5 (4.9)	59	0	NR
Wu et al. [50]	Retrospective	378	4.8	24 (6.4)	Surgery (87.3%)	NR	NR	Head (202) Body (54) Tail (115)	NR	44	NR	NR
Cohen et al. [51]	Retrospective	35	5.2	4 (11.4)	Surgery	NR	NR	Head (7) Body, neck (9) Tail (15)	NR	NR	0	NR
Guo et al. [52]	Retrospective	87	5.9	1 (1.1)	Surgery	29 (33.3) ^P^	0	Head (27) Neck (13) Body, tail (47)	0	46	4 (4.6)	NR
Karsenti et al. [10]	Retrospective	149	4.5	0	Surgery	NR	1 (0.67)	Head (63) Neck, body, tail (86)	4 (2.7)	33	78 (52.3)	NR
Wright et al. [53]	Prospective	78	4	0	Surgery	31 (39.7) ^Q^	0	Head (27) Body (18) tail (33)	1 (1.3)	36	44 (56.4)	0
Torres et a. [54]	Retrospective	16	6.3	0	Surgery	6 (37.5) ^R^	0	Head (16)	0	42	0	0
Hu et al. [55]	Retrospective	109	5.8	11 (10.1)	Surgery	NR	0	Head, neck (58) Body, tail (51)	0	62	0	NR
Won et al. [56]	Retrospective	46	3.9	0	Surgery	7 (15.2) ^NR^	0	Body (16) Tail (30)	0	35	0	NR
Hansen et al. [57]	Retrospective	15	5	1 (6.7)	Surgery	2 (13.3) ^S^	0	Head (6) Body (6) Tail (3)	0	40.3	NR	1 (6.7)
Liu et al. [58]	Retrospective	243	4.8	2 (0.8)	Surgery	NR	0	Head (64) Neck (27) Body (70) Tail (79) Others (3)	4 (1.6)	46	4 (1.6)	NR
Tjaden et al. [59]	Retrospective	52	4.4	1 (1.9)	Surgery	24 (46.1) ^T^	0	Body, tail (29) Others (23)	5 (9.6)	54	0	8 (15.4)
Ercelep et al. [60]	Retrospective	28	5.8	2 (7.1)	Surgery	NR	0	Head (8) Neck (2) Body, tail (18)	3 (10.7)	55.6	0	NR
Yang et al. [61]	Retrospective	113	4.8	1 (0.9)	Surgery	NR	NR	Head, neck (61) Body, tail (52)	4 (3.5)	49	0	0
Kumar et al. [62]	Prospective	50	7.7	2 (4)	Surgery	16 (45.7) ^U^	0	Head (33) Body, tail (17)	0	29	NR	NR
Zhan et al. [63]	Retrospective	91	6.9	1 (1.1)	Surgery	26 (28.6) ^V^	0	Head (32) Neck (14) Body, tail (45)	1 (1.1)	38	0	NR
Coelho et al. [64]	Retrospective	20	7.6	0	Surgery	6 (30) ^Y^	0	Head (6) Body (3) Tail (6) Body, tail (5)	0	38	0	NR
Sachan et al. [65]	Retrospective	13	6.5	0	Surgery	4 (30.8) ^Z^	0	Head (8) Body (3) Tail (2)	0	68	0	NR
Hanada et al. [66]	Retrospective	288	4.3	5 (1.7)	Surgery (96.5%)	NR	NR	Head (71) Body (96) Tail (84) Head, body (2) Body, tail (14) Unknown (21)	6 (2.1)	32	82 (28.5)	NR
Huffman et al. [67]	Retrospective	304	5.1	15 (5)	Surgery (93.7%)	NR	NR	Head (77) Body (40) Tail (132) Other (55)	NR	60	0	0
Wang et al. [68]	Retrospective	97	5.3	2 (2.1)	Surgery	41 (42.3) ^AA^	0	Head (37) Neck (12) Body, tail (48)	0	54	4 (4.1)	NR
Song et al. [69]	Retrospective	53	6.4	0	Surgery	14 (26.4) ^AB^	0	Head (15) Body (32) Neck (5) Extrapancreatic (1)	2 (4.2)	48	3 (5.7)	NR
Senthilnathan et al. [70]	Retrospective	17	7.5	0	Surgery	7 (41.2) ^AC^	0	Head, neck (6) Body, tail (11)	0	31	0	NR
Bhutani et al. [71]	Retrospective	11	6.9	0	Surgery	NR	0	Head (1) Body (1) Tail (6) Body, tail (2) Head, tail (1)	3 (27.3)	20	0	0
Bochis et al. [72]	Retrospective	13	8.2	0	Surgery	NR	1 (7.7)	Head (3) Body, tail (10)	0	18	0	0
Lima et al. [73]	Retrospective	13	8.8	0	Surgery	0	0	Head (5 Body (1) Body, tail (7)	0	83.8	0	0
Lubezky et al. [74]	Retrospective	32	5.9	1 (3.1)	Surgery	4 (12.5) ^AD^	0	Head (10) Body, tail (22)	3 (9.4)	49.2	22 (68.8)	NR
Beltrame et al. [75]	Retrospective	18	6.6	1 (5.6)	Surgery	5 (27.8) ^AE^	0	Head (3) Body (4) Tail (10) Head, body (1)	1 (5.6)	81.5	5 (27.8)	1 (5.6)
Marchegiani et al. [76]	Retrospective	131	4	1 (0.8)	Surgery	58 (44.3) ^AF^	0	Head (40) Body (37) Tail (44)	2 (1.5)	62	0	NR
Ugras et al. [77]	Retrospective	16	5.9	0	Surgery	NR	1 (6.2)	Head (6) Body, tail (10)	0	28	0	NR
Dai et al. [78]	Retrospective	45	6.3	0	Surgery	9 (20) ^AG^	0	Head (9) Neck (3) Body, tail (33)	1 (2.2)	51.7	0	NR
Zhang et al. [79]	Retrospective	62	7.2	3 (4.8)	Surgery	14 (22.6) ^AH^	0	Head (29) Body (12) Tail (21)	0	46	0	0
Serrano et al. [80]	Retrospective	32	4.7	2 (6.2)	Surgery	12 (37.5) ^AI^	0	NR	3 (9.4)	43	0	NR
Ren et al. [81]	Retrospective	19	6.3	0	Surgery	5 (26.3) ^AJ^	0	Head (6) Neck (1) Body, tail (12)	0	38.4	0	0
Kim et al. [82]	Retrospective	106	4.5	0	Surgery	42 (39.6) ^AK^	0	Head (32) Body (33) Tail (40) Multiple (1)	2 (1.9)	56.9	10 (9.4)	NR
Nakamura et al. [83]	Retrospective	14	4.8	0	Surgery	2 (14.3) ^AM^	0	Body (4) Tail (4) Body, tail (6)	0	29.5	0	NR
Yu et al. [84]	Retrospective	97	5.9	1 (1)	Surgery	31 (31.9) ^AN^	0	Head (20) Neck (9) Body, tail (68)	3 (3.1)	70.2	0	NR
Kang et al. [85]	Retrospective	351	5.7	4 (1.1)	Surgery	73 (20.8) ^AO^	1 (0.3)	Head (92) Body, tail (259)	9 (2.8)	158.7	0	NR
Cai et al. [21]	Retrospective	115	6.3	5 (4.3)	Surgery	22 (19.1) ^AP^	0	Head (41) Body (8) Tail (42) Neck, body (23) Head, tail (1)	2 (1.7)	58	0	NR
Estrella et al. [86]	Retrospective	64	6.6	5 (8)	Surgery	NR	NR	Head (19) Body (13) Tail (32)	2 (3.1)	76	0	NR
Zhang et al. [87]	Retrospective	28	6.4	0	Surgery	10 (35.7) ^AQ^	0	NR	1 (3.6)	39	0	NR
Wang et al. [88]	Retrospective	102	7	1 (1)	Surgery	34 (33.3) ^AR^	0	Head (34) Neck (19) Body, tail (49)	3 (3.4)	27	0	NR
El Nakeeb et al. [89]	Retrospective	24	9.2	0	Surgery	11 (45.8) ^AS^	0	Head (12) Body (2) Tail (10)	2 (8.3)	71.6	0	0
Wang et al. [90]	Retrospective	17	5.5	0	Surgery	5 (29.4) ^AT^	0	Head (5) Neck (2) Body, tail (10)	0	48.2	0	NR
Lin X et al. [91]	Retrospective	60	5.8	0	Surgery	22 (36.7) ^AU^	0	Head, neck (16) Neck (10) Body, tail (34)	1 (1.7)	47	0	NR
Suzuki et al. [92]	Retrospective	34	4.3	0	Surgery	NR	0	Head (16) Body (6) Tail (8) Body, tail (6)	0	67	0	NR

NR = not reported. ^A^ Pancreatic fistula (15), abscess (3), bleeding (1), delayed gastric emptying (2), bile leak (1), pancreatic insufficiency (3), reoperation (1). ^B^ Pancreatic fistula (119), delayed gastric emptying (4), hemorrhage (6). ^C^ Pancreatic fistula (6), hemorrhage (1), surgical site infection (1), pulmonary complication (1). ^D^ Pancreatic leak (4), abscess (2), chyle leak (2), ileus (1), other (2). ^E^ Pancreatic fistula (2), hemorrhage (1), abscess (2), chyle leak (4). ^F^ Pancreatic and chyle leak (11), wound infection (4), delayed gastric emptying (4), hemorrhage (1), intraabdominal collection (1), sepsis (1). ^G^ Pancreatic fistula (6), delayed gastric emptying (3), abdominal infection (13), bleeding (5), pancreatitis (2), multiple complications (4). ^H^ Pancreatic fistula (7), delayed gastric emptying (8), abdominal infection (9), bleeding (10), pancreatic insufficiency (50). ^I^ Pancreatic fistula (7), bleeding (1), abdominal infection (5), chyle or biliary fistula (4). ^J^ Pancreatic fistula (29), pancreatitis (9), abdominal infection (4), bleeding (3). ^K^ Pancreatic fistula (7), delayed gastric emptying (2), hemorrhage (3), pancreatitis (1). ^L^ Pancreatic fistula (2), others (3). ^M^ Pancreatic fistula (8). ^N^ Postoperative pancreatitis (24), delayed gastric emptying (7), fluid collection (4), new onset diabetes (4). ^O^ Pancreatic fistula (18), abdominal abscess (9), bleeding (2), gastric fistula (1). ^P^ Pancreatic fistula (21), abdominal infection (3), delayed gastric emptying (1), pancreatitis (3), hemorrhage (1). ^Q^ Pancreatic fistula (15), delayed gastric emptying (8), abdominal fluid collection (8). ^R^ Pancreatic fistula (3), infection (1), bleeding (1), pancreatitis (1). ^S^ Pancreatic fistula (1), bowel fistula (1). ^T^ Delayed gastric emptying (6), pancreatic fistula (8), abscess (1), bleeding (2), wound infection (3), chyle lead (4). ^U^ Pancreatic fistula (8), hemorrhage (1), delayed gastric emptying (2), chyle lead (3), abdominal collections (2). ^V^ Pancreatic fistula (21), bleeding (2), delayed gastric emptying (1), incomplete obstruction (1), pneumonia (1). ^Y^ Pancreatic fistula (3), surgical site infection (3). ^Z^ Pancreatic leak (2), surgical site infection (2). ^AA^ Pancreatic fistula (26), pancreatitis (5), chyle fistula (2), Delayed gastric emptying (2), abdominal infection (4), wound infection (1), abdominal pain (1). ^AB^ Pancreatic fistula (9), intraabdominal abscess (4), gastric fistula (1). ^AC^ Pleural effusion (2), pancreatic fistula (2), hemorrhage (2), chylorrea (1). ^AD^ Pancreatic fistula (2), wound infection (2). ^AE^ Pancreatic fistula (5). ^AF^ Pancreatic fistula (32), abdominal collection (20), pancreatitis (4), delayed gastric emptying (2). ^AG^ Pancreatic fistula (5), infection (3), delayed gastric emptying (1). ^AH^ Pancreatic fistula (6), abdominal abscess (4), wound infection (2), fluid collection (2). ^AI^ Pancreatic fistula (8), bleeding (1), intraabdominal abscess (2), wound infection (1). ^AJ^ Wound infection (1), pseudocyst (1), pancreatic fistula (3). ^AK^ Pancreatic fistula (32), abscess (4), delayed gastric emptying (4), bleeding (1), portal vein thrombosis (1). ^AM^ Pancreatic fistula (2). ^AN^ Pancreatic fistula (18), infection (8), delayed gastric emptying (4), bleeding (1). ^AO^ Pancreatic fistula (42), others (31). ^AP^ Pancreatic fistula (13), wound infection (2), fluid collection (3), pulmonary infection (3), gastrointestinal bleeding (1). ^AQ^ Pancreatic fistula (8), intraabdominal abscess (1), reoperation (1). ^AR^ Pancreatic fistula (16), thrombocytosis after splenectomy (16), gastrointestinal bleeding (1), pleural effusion (1). ^AS^ Pancreatic fistula (4), biliary leak (5), delayed gastric emptying (1), wound infection (1). ^AT^ Pancreatic fistula (3), pulmonary infection (1), pseudocyst (1). ^AU^ Pancreatic fistula (11), intraabdominal infection (6), delayed gastric emptying (2), respiratory complications (3). Not reported (118).

**Table 2:** Data summary of 70 papers (6651 patients).

	Cases	Papers	Patients	Value
Mean size of SPN (cm)	-	70	6651	5.8 cm
Metastasis	124	70	6651	1.9%
Treatment by surgery	6524	70	6651	98.1%
Morbidity after surgery	1163	49	3587	32.4%
Mortality after surgery	10	61	4415	0.2%
Recurrence	180	66	5910	3%
Follow-up, median, months	-	68	6615	51.7
EUS-FNA	356	66	6157	5.8%
Lesion ≤ 2 cm	75	20	1812	4.1%

**Table 3:** Details of adverse events.

	Cases	%
Overall adverse events	1163	-
Pancreatic fistula	559	48.1
Pancreatic leak and chyle leak	11	0.95
Bleeding or hemorrhage	46	3.9
Delayed gastric emptying	64	5.5
Pancreatitis	49	4.2
Wound or abdominal infection	73	6.3
Abscess or intraabdominal collections	80	6.9
Chyle, biliary leak, or fistula	26	2.2
Gastric fistula	2	0.17
Pancreatic insufficiency	53	4.6
Pulmonary complications	12	1
New onset diabetes mellitus	4	0.34
Ileus or incomplete obstruction	2	0.17
Bowel fistula	1	0.08
Gastrointestinal bleeding	2	0.17
Reoperation	2	0.17
Others	59	5.1
Not reported	118	10.1

**Table 4:** Metastatic SPN at diagnosis.

Citation	Site of Metastasis, (*N*)	Age	Size (cm)
Zou et al. [29]	Liver	NR	NR
Chen et al. [24]	Liver (4) Peritoneum (2) Abdominal cavity (2)	43	9.2
Lee et al. [26]	Liver (3) Omentum (1)	NR	NR
Wu et al. [50]	NR	NR	NR
Cohen et al. [51]	Liver (3) Peritoneal (1)	35.5	8.3
Guo et al. [52]	Liver	NR	NR
Hu et al. [55]	Liver (1) Spleen (6) Duodenum (3) Kidney (1)	NR	NR
Liu et al. [25]	NR	NR	NR
Yang et al. [46]	Liver (3)	NR	NR
Hansen et al. [57]	Liver	68	7
Liu et al. [58]	Liver (2)	NR	NR
Tjaden et al. [59]	Liver	65	7.5
Ercelep et al. [60]	Liver (2)	NR	NR
Yang et al. [61]	Liver	NR	NR
Kumar et al. [62]	Liver	NR	NR
Zhan et al. [63]	Liver	NR	NR
Hanada et al. [66]	Liver (3) Lung (1) Splenic vein tumor thrombus (1)	57.4	9.5
Huffman et al. [67]	NR	49	12.5
Wang et al. [68]	Liver (1) Peritoneum (1)	NR	≥5
Lubezky et al. [74]	Liver	NR	8
Beltrame et al. [75]	Liver	21	8
Marchegiani et al. [76]	Liver	NR	NR
Zhang et al. [79]	Liver	NR	NR
Serrano et al. [80]	Liver	38.5	NR
Cai et al. [93]	Liver	NR	NR
Yu et al. [84]	Liver	NR	NR
Kang et al. [85]	Liver	NR	NR
Estrella et al. [86]	Liver (3) Peritoneum (1) Omentum (1)	20.2	10
Wang et al. [88]	Liver	NR	NR

NR = not reported.

**Table 5:** Demonstrates the studies on EUS-RFA in SPN.

Citation	Study	Patients	Size of SPN (mm)	Metastasis	Treatment	Morbidity After RFA	Mortality After RFA	Location, N	Recurrence	Follo-Up, Median, Months	EUS-FNA, *N* (%)	Lesion ≤ 2 cm, *N* (%)
Napoleon et al. [95]	Case series	3	16.7	0	RFA	0	0	Head (3)	0	9	3 (100)	3 (100)
Choi et al. [97]	Case report	2	21.5	0	RFA	0	0	Head (1) Tail (1)	0	28	0	1 (50)

## Data Availability

The study materials are available from the corresponding author upon reasonable request.

## Abbreviations

CT: computed tomography; EUS-RFA: endoscopic ultrasound-guided radiofrequency ablation; SPN: solid pseudopapillary neoplasm; EUS-FNA: endoscopic ultrasound-guided fine needle aspiration.
